# MicroRNAs in Molecular Classification and Pathogenesis of Breast Tumors

**DOI:** 10.3390/cancers13215332

**Published:** 2021-10-23

**Authors:** Vinitha Richard, Matthew G. Davey, Heidi Annuk, Nicola Miller, Róisín M. Dwyer, Aoife Lowery, Michael J. Kerin

**Affiliations:** Discipline of Surgery, The Lambe Institute for Translational Research, National University of Ireland, H91 YR71 Galway, Ireland; m.davey7@nuigalway.ie (M.G.D.); heidi.annuk@nuigalway.ie (H.A.); nicola.miller@nuigalway.ie (N.M.); roisin.dwyer@nuigalway.ie (R.M.D.); aoife.lowery@nuigalway.ie (A.L.)

**Keywords:** microRNAs, breast cancer, molecular classification, intrinsic subtypes, hormone receptors, cancer stem cells

## Abstract

**Simple Summary:**

Breast cancer is an ideal model of a heterogeneous disease that is triggered by genetic changes in the normal mammary epithelial cells and manifest as variants of breast tumor subtypes in individuals. Advancement in molecular and genomic profiling techniques, in particular the microRNA profiling have improved the ambiguity related to the presence of multiple breast tumor subtypes. This review discusses in detail, the efficient categorization of breast tumor subtypes based on expression of microRNAs and also highlights the significant role of microRNAs in regulating both the tumor cells and the host microenvironment in driving tumor initiation, progression, chemoresistance and eventual spread of the disease. MicroRNAs may be rightfully deemed as excellent biomolecules deserving a detailed investigation.

**Abstract:**

The current clinical practice of breast tumor classification relies on the routine immunohistochemistry-based expression analysis of hormone receptors, which is inadequate in addressing breast tumor heterogeneity and drug resistance. MicroRNA expression profiling in tumor tissue and in the circulation is an efficient alternative to intrinsic molecular subtyping that enables precise molecular classification of breast tumor variants, the prediction of tumor progression, risk stratification and also identifies critical regulators of the tumor microenvironment. This review integrates data from protein, gene and miRNA expression studies to elaborate on a unique miRNA-based 10-subtype taxonomy, which we propose as the current gold standard to allow appropriate classification and separation of breast cancer into a targetable strategy for therapy.

## 1. Introduction

Female breast cancer is the most commonly diagnosed cancer, accounting for 11.7% of all cancer burden cases, and is the leading cause of cancer-related death worldwide [1]. Breast tumor heterogeneity, detectable from tumor histology and clinical outcomes, has led to the development of pathology-driven and molecular-based disease classification [2]. Breast cancer (BC) is primarily classified based on the differential expression of cell surface protein hormone receptors (HR)—estrogen or progesterone receptors (ER; PR) and human epidermal growth factor 2 (ERBB2; formerly HER2)—and is categorized into three major subtypes: ER+/PR+/ERBB2- (70% of patients), ERBB2+ or HER2+ (15–20%) and triple-negative breast cancer (TNBC or ER-/PR-/ERBB2- (15%)) [3]. With the advent of genomic microarray and sequencing technologies, breast tumors have been re-categorized based on the expression of 50 genes, the PAM50 (Prediction Analysis of Microarray 50) gene classification, to five major intrinsic subtypes (Luminal A (LumA/LA), Luminal B (LumB/LB), HER2-enriched, Basal and Normal-like group) [4]. Several additional classifications using mathematical Topological Data Analysis (TDA gene expression signature) led to the proposition of seven breast tumor subtypes: (Basal/HER2, LumB/Basal, LumA, Basal/Myoepithelial (Myo), Myo/LumA, Myo/LumB and Myo/LumB/HER2) [5]. Molecular profiling of the aggressive basal tumor subtype revealed the presence of five or six additional subclasses, each with its own molecular features and sensitivity to standard chemotherapy [6]. The integration of data on copy number alterations (CNAs), methylation pattern and its prognostic relevance in breast cancer led to the re-grouping of intrinsic subtypes to ten integrated clusters (IntClust or (ICs)1-10) [7,8,9]. The revelation of microRNA (miRNA) signatures from high-throughput sequencing experiments on BC samples from various case studies, the METABRIC study (Molecular Taxonomy of Breast Cancer International Consortium) [7,8], TCGA (The Cancer Genome Atlas) [10] and SCAN-B (The Sweden Cancerome Analysis Network—Breast initiatives) [11,12], have further led to the systematic categorization of ten new molecular subtypes of breast cancer (Basal; Basal-HER2; Basal-LumB; Basal-LumA; HER2; HER2-LumB; HER2-LumA; LumA-LumB; LumA and LumB), thus reinforcing the concept of a novel molecular taxonomy in breast cancer [11,12,13,14,15,16,17]. Even amidst different subtypes with similar genomic and clinical profiles, the cancer survival rates vary, highlighting the need for functional biomarkers that improve risk stratification and advocate treatment options as well. Highly sensitive, breast-tumor-specific biomarkers for early diagnosis both in tissue and in circulation and for tracking remission at different time points of treatment regimen represent a major current deficit.

Micro-ribonucleic acids (microRNAs/miRNAs/miRs) are ~22-nucleotide-long RNA molecules that act as key post-transcriptional regulators of primary malignant transformational events such as gain- and loss-of-function gene mutations, dysregulated gene expressions and epigenetic regulations [18,19]. The discovery of novel miRNAs in the body fluids (serum and plasma) of breast cancer patients has highlighted their use as non-invasive biomarkers of disease [3,20]. Despite the fact that most circulating miRNAs in cancer patients may not originate from tumors but rather reflect the host homeostatic response, the systemic miRNAs do have the potential to be used in monitoring disease progression and predicting long-term risk. Dysregulation of microRNAs is also an early event in tumorigenesis, most significant during the transition from normal to atypical ductal hyperplasia (ADH), thereby marking them as promising cancer biomarkers, targets and effectors for the early diagnosis of cancer [17,21,22]. MicroRNA expression profiles alone or integrated with mRNA profiles are reported to improve breast cancer subtyping and are even more informative than protein-coding RNAs [22]. The expression patterns of certain miRNAs are found to be associated with prognosis for the long-term risk of relapse (ROR), long-term breast cancer survival and also improves the risk stratification in BC patients [23,24,25].

Bench to bed-side translation of advanced genomic technologies, with the key focus on accurate diagnosis and stratification of breast cancer patients for treatment optimization, have led us to raise the following questions: (i) Are microRNAs capable of differentiating the cellular compartments consisting of the cell-of-origin, the normal mammary stem cells and the stem-like cancer cells from the bulk of transformed epithelial cells within the breast tissue hierarchy? (ii) Could microRNAs be deemed as comprehensive molecular determinants of breast tumor subtypes? (iii) Is the miRNA expression pattern predictive of the stages of tumor progression and disease-free survival in breast cancer patients? (iv) Are the breast tumor tissue-specific and systemic microRNAs capable of modulating the tumor stromal microenvironment to enhance the survival of cancer cells? This review attempts to update the knowledge base on miRNA signatures from recent high-throughput sequencing studies that elucidate miRNAs as predictors of additional breast tumor tissue subtypes and as trackable serum biomarkers with a regulatory role as modulators of tumor progression and the potential of being utilized as druggable targets.

## 2. MicroRNAs as Determinants of Breast Tissue Heterogeneity

### 2.1. Tissue Hierarchy of Normal Mammary Gland

**Figure 1:**
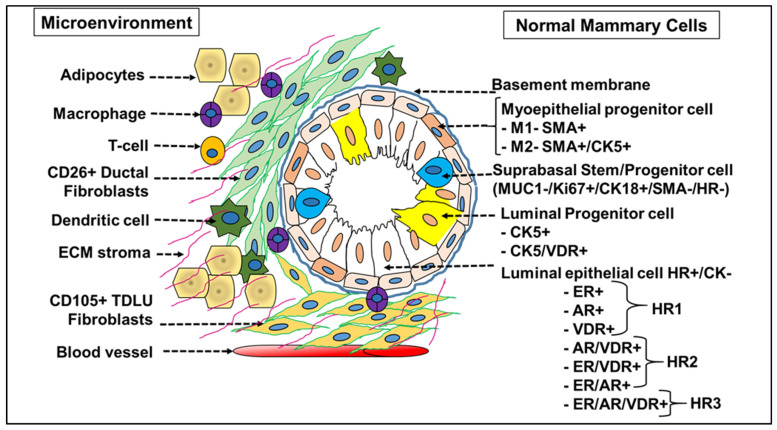
Cellular landscape of normal breast tissue. A normal breast is made up of lobules with varying expression levels of Cytokeratin 5 (CK5) and vitamin D receptor (VDR). Major lobular types display the phenotype of high ER/AR/VDR (HR+), low Ki67 and CK5, while the second lobular type is dominated by VDR+ and VDR/CK5+(HR+) expressing cells with less proliferative Ki67+ cells, indicating the presence of a differentiated set of cells within lobules of the same normal tissue. Normal human breast tissue also stained positive for Keratins (CK) 5/14/17 in cells facing the lumen, above the CD10/SMA+ myoepithelial (basal) layer in the interlobular ducts and in the luminal layer of lobules, specifying that they are markers of luminal epithelial cells. ER+ and CK5+ staining are bimodal (biphasic), with ER+ cells observed to be more differentiated derivatives of transit-amplifying (TA) or progenitor CK5+ luminal cells in the normal epithelial cell hierarchy. Normal luminal cells exist in 11 differentiation states and are grouped into 4 major hormonal subtypes (HR0, HR1, HR2 and HR3) based on expression of vitamin D, androgen and estrogen hormone receptor (HR).

The human mammary gland is a highly heterogeneous organ and is equally dynamic, with multiple stages of remodeling during the developmental stages and in the transformation from benign to malignant phase [2,26]. The cellular heterogeneity of normal mammary and tumor tissue is complex, hindering the accurate identification and categorization of cell types. The tissue homeostasis is orchestrated by an array of mammary stem cells (MaSCs), progenitors and differentiated cells. The diverse nature of breast cancer is linked to the cells-of-origin and the selection of clones that thrive despite an accumulation of genetic mutations triggering the initiation of pre-cancerous lesions. A deeper understanding about the cells-of-origin, the evolution of multiple tumor subtypes expressing diverse hormone receptors and the collective molecular mechanisms driving the disease progression would unveil novel targets and treatment options for personalized medicine. Insights from normal mammary gland biology and the differentiation states of cell fractions in normal breast tissue provide a reference classification system for breast tumors (Figure 1). The distribution of specialized cells across different tissue layers with varying sensitivities and resistance to cancer causing stimuli results in tumor clones with different clinical behaviors [27]. 

### 2.2. Cell Subtypes of Normal Breast and Tumor Tissue

**Figure 2:**
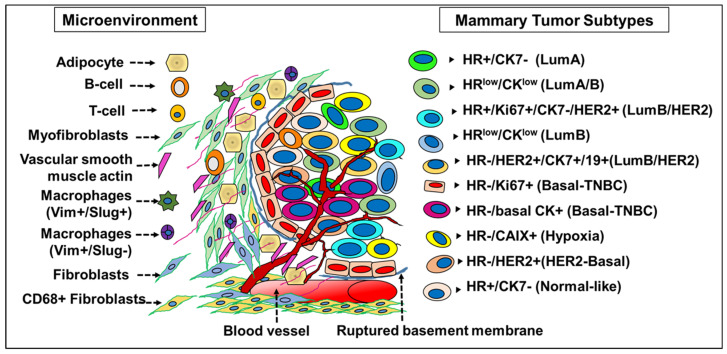
Molecular taxonomy of breast tumor subtypes. In tumor tissue, de-differentiation leads to multiple cell phenotypes based on differential expression of ER/PR/HER2 hormone receptors and cytokeratins. Molecular profiling combined with mutations and copy number aberrations of tumor tissue revealed 10 novel molecular subgroups, which are also representatives of luminal subtypes found in normal breast tissue. The presence of minimal levels of proteins despite higher levels of mRNA often leads to miscategorization between the luminal and myoepithelial cells. This discrepancy is indicative of post-transcriptional repression of protein expression by the microRNAs, which in turn underlies the heterogeneity in cellular protein expression in breast cancer. Accuracy of tumor categorization is seemingly precise at the miRNA level as it enables early detection of malignant transformation both in situ and in circulation and divides tumors into 10 subtypes with potential implications in precision medicine.

A detailed screening of epithelial markers in normal breast cells led to the identification of 11 differentiation states in normal luminal cells (L1–L11 groups) based on the expression of hormone receptors (HR)—vitamin D (VDR), androgen (AR) and estrogen receptor (ER). Successively, the tumors generated also correspond to four subtype categories depending on the expression profile of these three receptors: (i) HR0—(VR-/AR-/ER-), (ii) HR1—(VR-/AR-/ER+), (iii) HR2—(VR-/AR+/ER+) and (iv) HR3—(VR+/AR+/ER+) [23]. The HR classification differed from the classical ER/PR/HER2 scheme and actually predicted prognosis, with the HR3 subtype reporting the best outcome and the subtype HR0 reflecting the worst prognosis [27]. The emergence of phenotypically different luminal tumor subtypes (from the normal luminal L1-L11 subtypes, wherein L1 and L2 are grouped together) is due to the presence of an underlying epigenetic regulatory mechanism that is unique to the cell-of-origin [27]. This fated transformation of a HR precursor cell to a HR+/- tumor subtype is not due to the mutations or amplifications in the genes coding for HR proteins (ER, AR or VDR), which are relatively rare events in breast cancer [27]. ER+ mammary cells are also adept in stimulating proliferation in ER- cells through the secretion of growth factors and mediated by transcriptional regulators, mainly microRNAs [28]. Independent data generated from the screening of miRNA clusters have validated the presence of 10 different breast tumor subtypes in accordance with the normal mammary epithelial counterpart [12] (Figure 2). To garner a better understanding of the role of microRNAs in breast tumors, it is imperative that we understand the significance and the role of a specific miRNA expression pattern in normal breast tissue stem cells and its differentiated tumor cell counterpart.

### 2.3. Cells-of-Origin and microRNAs as Regulators of Stemness and Cell Fate

Failure of treatment modalities leading to recurrence in breast tumors has been attributed to the presence and survival of a small fraction of tumor cells that retain the properties of adult stem cells and the potential to regenerate a whole new tumor [29]. Stem-like cancer cells that express the (CD44+CD24−/lin−) cell surface marker phenotype and share the molecular regulatory features of normal mammary stem cells (MaSCs) are termed breast cancer stem cells (BCSCs) [29,30]. The differential expression of miRNAs and miRNA clusters located on distinct chromosomal regions also provides an additional layer of regulatory control on oncogenes during the malignant transformation of cells [30]. Three miRNA clusters (miR-200c-141, miR-200b-200a-429 and miR-183-96-182) are proven to be downregulated in human BCSCs and the proto-oncogene *BMI1* is a validated target of miR-200c [30]. MicroRNAs are also capable of regulating the distinct inter-cell state transitions, epithelial–mesenchymal transition (EMT) and mesenchymal–epithelial transition (MET), in both normal and malignant breast stem cells, thus facilitating the co-existence of multiple stem cell states, interpreted as biological heterogeneity in tumors [31]. 

Since the first report by Lee et al., deciphering the role of microRNAs in the developmental stages of an entirely different species, Caenorhabditis elegans (c. elegans) [32], translational research has attributed these small endogenous microRNAs with additional potential as clinical biomarkers for the early detection of breast tumors [33], as predictors of outcomes of treatment and surgery [34], and they are also associated with the risk of distant metastasis [35]. The biogenesis of microRNAs is initiated in the nucleus, from the non-coding regions of DNA harboring the miRNA genes, which undergo transcription to generate 1000-nt-long poly-adenylated primary miRNA transcripts (pri-miRNAs) [36]. These are further cleaved to a 70–90-nt-long precursor miRNA (pre-miRNA) by the RNase type III enzyme Drosha and its complementary binding partner DCGR8 and are transported out of the cellular nucleus as hairpin structures by the export protein Exportin 5 [37]. In the cytoplasm, the pre-miRNAs are then subjected to enzymatic cleavage by RNase III Dicer to generate a miRNA duplex [38]. One strand of this short-lived duplex represents a functional (19–25-nt-long) mature microRNA strand, which is incorporated into the miRNA-associated RNA-induced silencing complex (miRISC) [39,40]. The miRNA–RISC complex targets and binds to the 3′ or 5′ untranslated regions of target messenger RNA (mRNA) with complementary sequences to the mature miRNA and directly promotes the mRNA degradation or inhibition of translation to proteins [33,40]. The role of miRNAs in dictating the cancer phenotype by acting either as an oncogene or as a tumor suppressor needs to be carefully scrutinized [41]. 

Differential expression of microRNAs also enables the clear distinction of cell types evidenced by high expression of miR-let7c, miR-125b, miR-126, miR-127-3p, miR-143, miR-145, miR-146-5p and miR-199a-3p in normal mammary epithelial basal cell types, whereas miR-200c and miR-429 are upregulated specifically in the luminal cell type [42]. MicroRNA profiling experiments revealed consistent low expression of three clusters, the miRNA-200c-141 cluster, the miR-200b-200a-429 and the miR-183-96-182 cluster, in human BCSCs as compared to mature epithelial cells [30]. Diminished expression of miR-200c inversely elevated the expression of *BMI1* gene, *ZEB1* and *ZEB2* (the two transcriptional repressors of E-cadherin), thereby enhancing the self-renewal, differentiation and EMT pathways, respectively, that are crucial in the maintenance of normal MaSCs and tumorigenic BCSCs alike [43,44]. Elevated expression of miR-199a is another shared trait of both normal MaSCs and CSCs that protects stem cells from differentiation and senescence by directly repressing nuclear receptor corepressor LCOR, which primes interferons (IFNs) secreted by epithelial and immune cells in the mammary gland [45]. MiR-221 acts as a dual regulator of cellular hierarchy in normal breast tissue, with overexpression in the stem-like normal myoepithelial cells and in the stem-like cells of malignant luminal types of cancer, wherein it specifically targets the *ATXN1* gene related to EMT and is also associated with poor clinical outcomes [46]. Differentiated tumor cells forming the bulk of breast tumors often regress post-therapy, but microRNAs effectively mediate self-renewal and differentiation amongst the tumor-initiating cells (T-ICs), enhancing their survival and the risk of relapse in patients. 

The majority of breast cancer cells reflect a unified miRNA signature of significantly reduced expression of miR-10b, miR-125b, miR-145 and high expression of miR-17–5p, miR-29b–2, miR-181b–1, miR-146, miR-21 and miR-155 [47,48]. MicroRNA profiles of inflammatory breast cancer (IBC) samples confirmed the upregulated expression of miR-221, miR-222, miR-18, miR-106B and miR-20 and downregulated expression of stem cell-specific miR-141, miR-200a, miR-200b, miR-200c, miR-205, miR-335, Let-7 and miR-429 [49,50]. Overexpression of miR-221 and miR-222 in IBC is also associated with resistance against endocrine therapy in breast cancer [51]. MicroRNA-31 functions as a pro-oncogenic miR and is identified as the key regulator of MaSC activity that promotes mammary epithelial proliferation and MaSC expansion in vivo [52]. Reduced expression of miR-31 in tumors compromised the number of cancer stem cells and decreased the tumor-initiating ability and lung metastasis [52]. Another microRNA, miR-206, is highly expressed in normal MaSCs and acts as a tumor suppressor by inducing G1-S cell cycle arrest, leading to reduced cell proliferation and EMT in MaSCs [53]. Akin to miR-206, miR-205 also acts as a tumor-suppressing epigenetic regulator that determines the fate of MaSCs, limits the cell proliferation and symmetric expansion of MaSCs and reduces differentiation via EMT [54]. Activation of miR-205 led to diminished stemness potential in breast cancer cells, implying its feasibility as a therapeutic target [54]. The overexpression of miR-93 promoted cellular differentiation and also determined the fate of normal and malignant MaSCs by downregulating the expression of multiple stem cell regulatory genes [55]. The miR-424/503 cluster, reportedly targeting the LRP6 co-receptor, works in tandem to regulate the mammary epithelial stem cell fate dictated by ovarian cycles and also drives tumorigenesis through modulating the canonical Wnt signaling [56]. The impact of an individual microRNA in regulating the expression of multiple gene targets occurs within the stem cell pool (e.g., miR-93 targets *JAK1*, *STAT3*, *AKT3*, *SOX4*, *EZH1* and *HMGA2* genes) [55] and in the de-differentiated tumor cells (e.g., miR-210 targets *VEGF* and *RUNX3* gene; and miR-193 family targets *CCND1*, *PTEN*, *ER* gene) [57]. This eventually alters the fate of the cell type, signifying the utility of microRNAs as a potent genomic tool to impede the rise and spread of malignant cancer cells. 

The fate of stem cells in mammary tissues and the maintenance of homeostasis are hitherto known to be controlled by the master regulators, the Homeobox genes [58]. Recent studies have indicated that microRNAs in turn act as the primary controllers of the master regulators, with the expression of the miR-196 family members clearly targeting and suppressing the expression of the *HOXC8* gene in breast cancer stem/progenitor cells across all molecular subtypes of breast cancer, leading to the conclusion that *HOXC8* is a tumor suppressor gene target [58]. Loss of *HOXC8* gene function in non-tumorigenic mammary epithelial cells increased the self-renewal activity, expanded the cancer stem/progenitor cell pool, prevented retinoic acid (RA)-induced differentiation and directed transformation in the mammary gland lineage [58]. Similarly, overexpression of a tumor suppressor, miR-489, led to the reduced expression of the CD49f^hi^CD61^hi^ mammary progenitor cell population, inhibited tumor growth, delayed HER2-induced tumor initiation and lung metastasis [59]. Low expression of miR-140 is observed in ERα-/basal-like ductal carcinoma in situ (DCIS) stem-like cells in comparison to normal stem cells and is reported to directly target the most significantly activated stem-cell factors, *SOX9* and *ALDH1* [60] (Table 1). MicroRNAs may act as both suppressors and promoters of oncogenes, with a definitive function depending on the fraction of cells (normal or cancerous) in which it is expressed. Likewise, miR-34a functions as a tumor suppressor that can target up to nine upstream regulators and also induce differentiation to luminal-like cell types when overexpressed in triple-negative mesenchymal-like cancer cells (enriched in CSCs) [61]. Therefore, induced activation of tumor suppressor miRs may aid in the targeted eradication of CSCs that respond poorly to conventional therapies and also promote mammary epithelial cellular plasticity.

### 2.4. MicroRNAs as Determinants of Normal from Hereditary Breast Tumors

Distinct microRNA signatures specifically characterized hereditary breast cancer (HBC), sporadic breast cancer (SBC) and HBCs of unknown genetic origin (also termed ‘BRCAX) from normal breast tissues (NBT) that are also wild-type/carriers/non-carriers of germline pathogenic variants of tumor suppressor genes, breast cancer type 1 and 2 (*BRCA1* and *BRCA2*) susceptibility genes [62,63]. Gene interaction network modeling linked BRCA1 mutations to the overexpression of insulin-like growth factor receptor-1β (IGF-R1β), which in turn led to the overexpression of HER2 and epithelial growth factor receptor (EGFR) proteins [64]. This also rationalizes the reported sensitivity of early-stage or a metastatic subset of HER2+ BRCA+ patients to targeted therapy by Trastuzumab and provides an alternative means of detecting BC samples harboring BRCA mutations through miRNA signatures [64]. A minimum of 20 oncogenic miRNAs that promote the features of proliferation, angiogenesis, invasion, migration and more than 30 tumor-suppressive miRNAs or cluster families that play a role in the negative control of cell proliferation, migration and cell apoptosis are detected to be transcribed together and co-expressed in breast tumor cells [60]. The ability of microRNAs to target multiple genes and regulate gene expression prior to translation has opened new avenues of a prospective understanding of breast cancer in the context of pre-diagnosis, patient stratification, predicting response to therapy and disease-free survival. 

## 3. MicroRNA-Based Molecular Taxonomy of Breast Tumor Subtypes

The mathematical topological data analysis (TDA) method executed on three breast cancer sample sets—1082 samples of TCGA datasets, 1904 samples of METABRIC, PAM50 mRNA expression datasets and 290 GTEx normal breast dataset—also led to the proposal of multiple tumor subtypes [9,10,11,12,13,14,15,16,17]. The revelation of the existence of multiple overlapping subtypes in addition to the PAM50 gene expression-based five-subtype categorization and the distinct miRNA profile of these subgroups calls for a reanalysis of the interactions between these microRNAs and the tumor milieu in governing disease pathogenesis. Breast epithelial tumor cell phenotypes presented a distinctive enrichment pattern consistent with the previously defined luminal cell types (L1/L2–L11) in normal mammary tissue, providing the basis of this new molecular nomenclature [27]. Alternative methods of tumor classification focused on clustering the tumor samples on the basis of mutations in the PAM50 list of genes and by mapping the chromosomal aberrations. This in turn led to the proposal of ten integrated clusters (IC 1-10), which also confirmed the presence of 10 tumor subtypes with an integral set of mutated genes [14]. The IC-5, 10 and 2 clusters represented tumor subtypes with poor prognosis, whereas subtypes in categories (IC-9, 6 and 1) displayed intermediate prognosis. Good prognosis is the highpoint of the subtypes of IC-8, 4, 7 and 3, which are also related to the HR2 and HR3 categories of normal luminal subtypes. 

A successive phenogenomic study integrated both the single cell proteomics approach and gene expression profiling on breast tumor tissue arrays and the data output grouped the individual cancer cells as distinct phenotypes and the whole breast tumors as a cluster of phenotypes [16]. Interlinking the transcriptomic data with the tumor cell phenotype and clustering of samples based on the similarity of expression also independently confirmed the presence of multiple breast tumor subtypes (Table 2). LumA/(HR+) breast tumor cells are represented by the protein phenotypes (31, 48, 53) and the integrative clusters (IC-3, 4+, 6, 7 and 8) [16]. More proliferative LumB tumors displayed a combination of phenotypes, [HR+Ki67+, phenotype 33] and [HR+, phenotype 31] respectively. HR- epithelial tumors with ERBB2 amplification or high expression of HER2 displayed phenotype 16. The luminal cell [ER-/low/CK7/19high] phenotype (phenotype 46) is rather similar to the HER2+ and IC-3 tumors [16], signifying that these tumors may have originated from cells of different mammary lineages [65]. Though the knowledge from these high-throughput datasets concerning the presence of multiple tumor subtypes seems promising, the translational aspect is meagre owing to the requirement of expertise and advanced techniques. 

A contemporary study focusing on the small RNA sequencing of 186 breast tumor samples from the SCAN-B initiative identified 684 microRNAs per sample, which, upon clustering, led to the identification of signatures of differentially expressed miRNAs associated with 10 intrinsic subtypes (HER2, HER2-Basal, HER2-LumA, HER2-LumB, Basal, Basal-LumA, Basal-LumB, LumA, LumB and LumA-LumB) [12] and 73 unique microRNAs related to four well-defined clusters that segregated the ER+ luminal tumors from ER- Basal-like and most HER2-enriched tumors [12]. The miRNA cluster-1 presented high expression of mir-26, mir-5681a, mir-5695, mir-887, mir-149, mir-375, mir-342, mir-29c, mir-29b, mir-499a and mir-190b [66] and downregulated expression of mir-455-3p, mir-934, mir-135b and mir-5 that featured as the miRNA cluster-2 in ER+ tumors [12]. ER- tumors are characterized by inverse expression levels of miRs in ER+ tumors and also the overexpression of mir-18a (miR-18a-5p) (cluster-3), which repressed the expression of ERα directly by binding to its mRNA [67]. The PAM50 categorization is only partially effectual in the stratification of clinically determined HER2+ tumors, whereas the HER2-enriched subtype is mainly defined by the high expression of miRNA genes: mir-34a, mir-2115, mir-4728 and mir-7158 [12,68]. Mir-4728 is a poorly conserved microRNA with low expression in most normal tissues, but is encoded within the HER2 oncogene and is co-amplified in HER2+ breast cancer [12,68]. HER2+ tumors that are also ER+ (Luminal B tumors) have a distinct miRNA profile (miRNA cluster-3). The miRNA cluster [mir-99a/let-7c/mir-125b-2] acts as a candidate tumor suppressor significantly upregulated in LumA tumors compared to LumB, making it an ideal prognostic marker of the LumA subtype associated with the regulation of inflammation and stem-like properties [12,69]. 

As breast tumor progresses, expression of VDR is lost, implicating a profound role in breast tumor initiation [70]. Discrepancy between the levels of VDR proteins and mRNA led to the postulation of miRNA-mediated post-transcriptional regulation or mRNA degradation [70]. The 3’ untranslated region of human VDR mRNA apparently harbors the miR-125b recognition element (MRE125b), strongly indicating that endogenous VDR levels are repressed by miR-125b [70]. Contradictory studies also suggested fluctuations in the expression levels of VDR and miR-125b, evidenced by the reduced expression of miR-125b and high expression levels of VDR in advanced breast cancers [71]. As an incidental finding that validates the normal mammary epithelial HR groups in Santagata S. et al. in [27], Huss et al. performed the nuclear and cytoplasmic staining of tumor tissue microarrays (TMA) and reported that VDR+ expression is highest in the order LumA > LumB > HER2 and the lowest in TNBC [72]. Moreover, nuclear VDR positivity in LumB-like tumors is found to be significantly associated with a decreased risk of breast cancer death and seemingly resembled the HR3 positive groups (LA/LB) with good prognosis [72]. Thus, changes in the levels of miR-125b is an indirect predictor of VDR expression levels and its subsequent role in the risk stratification of breast cancer patients.

To sum up, the complexities arising with subtype categorization originating from the transformation of various cell types in the normal epithelium to the integration of the mutational profile, an overlay of transcriptomic gene expression signatures, protein level phenotyping and whole tumor tissue mapping of the cellular distribution of these biomarkers have, in conclusion, highlighted the same set of 10 molecular breast tumor subtypes, which are also easily detectable at an earlier stage of transformation, by relying only on the microRNA profile (Table 3). If this holds true, diagnosis and treatment strategies may possibly be modified by merely tracking changes in miRNA expression levels. Though this finding is a breakthrough, extensive validation is yet to be performed both at the tissue level and in circulation to enable the translation of this finding to a clinical setting.

## 4. MicroRNAs Predicting Treatment Resistance and Survival

Despite the presence of multiple clinical-genomic risk classification tools, women with apparently similar risk profiles vary in their rates of risk of relapse (ROR), response to treatment and disease-free survival (DFS). Independent biomarkers such as microRNAs in cohorts of long-term follow-up (>15 years) may improve risk stratification in an intermediate-to-high risk of relapse group [23]. Overexpression of miR-210 represented an aggressive phenotype associated with worse prognosis in multiple cancers [73]. In breast cancer patients, miR-210 represented a very high-risk subgroup and miR-29c recognized a low-risk group in the ROR-PT medium-risk category [23]. Even though the miR-29 family acted both as a tumor suppressor and a promotor of breast cancer [74], higher levels of miR-29c are also linked to worse prognosis [23]. Higher levels of miRNA-187-3p and miR-143-3p and lower levels of miR-205-5p are also associated with shorter survival times in breast cancer [23,75]. Reproducible expression levels of these miRs across different breast tumor subtypes post-neo-adjuvant chemotherapy (NAC) and the subsequent risk stratification are essential to clinically approve the utility of miRs as predictors of ROR and survival.

Induced therapeutic resistance to numerous drugs used against ER+ breast cancer cells arises predominantly as a consequence of the activation of mitogen-activated protein kinases (MAPKs) and mitogen-activated protein kinase phosphatases (MKPs), which are involved in resistance to tamoxifen, doxorubicin, paclitaxel, proteasome inhibitors and oxidative-stress-induced cell death [76]. Overall, 30% of ER+ tumors are non-responsive to tamoxifen at the outset of treatment due to de novo (or acquired) resistance [77]. Similarly, the activation of EGFR and HER2 also appears to facilitate the growth of tamoxifen-resistant tumors partly through non-genomic ER mechanisms [68,78]. A patient-derived miRNA-based MAPK hyperactivation (hMAPK) signature (hsa-miR-221, hsa-miR-222, hsa-miR-130a, hsa-miR-31, hsa-miR-27a, hs-miR-23a and hsa-miR-22) correlated with ER- breast tumors having a high stromal score and poor clinical outcomes evidenced by decreased recurrence-free survival (RFS) and disease-specific survival (DSS) [79,80]. Paradoxically, this hMAPK-miRNA signature (especially miR-221/222) is also highly expressed in conditioned media (CM) from cancer-associated fibroblasts (CAFs) of ER-/Basal breast tumors (Basal CAFs) and represses the expression of both ER protein and mRNA in ER+ tumors in a paracrine manner [81]. A decrease in the expression of miR-489 highlights enhanced chemoresistance, an increase in tumor size, advanced pTNM stage, lymph node metastasis and poor prognosis in breast cancer [82]. *SPIN1, VAV3, BCL2* and *AKT3* are found to be direct gene targets of miR-489, and elevated expression of *SPIN1* is observed in drug-resistant, metastatic breast cancer tissues with a (PR+) HR status. Either the inhibition of *SPIN1* or overexpression of miR-489 is effective in suppressing the PI3K-Akt signaling pathway genes (*PIK3CA, AKT, CREB1* and *BCL2)*, which are also considered downstream effectors of *SPIN1*. This study implies that inducing the expression of miR-489 could reverse the chemoresistance of breast cancer by targeting *SPIN1* and the PI3K-Akt pathway [82]. This inter-connected regulatory network clearly indicates that a variation in the levels of microRNAs could direct the re-emergence of resistant tumor cells post-chemo- and hormonal therapy.

A second inherent means of acquired resistance to targeted therapy trastuzumab (Tzb), actively deployed by Tzb-resistant (Tzb-R) HER2+ BC cells, is an upsurge in the rate of autophagy [83]. Autophagy is a cytoprotective mechanism adapted by both normal and tumor cells to increase cell survival during states of metabolic stress, hypoxia and chemotherapy-induced cell death [84]. The molecular mechanism that regulates autophagy is attributed to a tumor suppressor microRNA, miR-567, that is significantly downregulated in Tzb-R breast cancer [85]. Higher expression of miR-567 in turn suppressed the expression levels of the target gene *ATG5*, which inhibited autophagy, sensitized patients to treatment and resulted in better survival than non-responders [86]. MiR-567 is also capable of propagating Tzb resistance amid heterogeneous breast cancer cells by incorporation into exosomes [85]. Tumor cells activate the mechanisms of autophagy and exosomal microRNA-mediated regulation as part of acquired resistance or as a consequence of changes in the cellular phenotype, often well-recognized after chemotherapy treatments, thereby limiting the therapeutic options to salvage the situation. Several of the functional targets of miRs are yet to be elucidated in breast cancer, providing little or no detail as to the mechanisms of action that drive tumor pathogenesis. In addition to the potential of microRNAs to intercede tumor cell state transitions and acquired resistance, it also aids in the adaptation of cells in the tumor vicinity to the changing tumor microenvironment (TME).

## 5. Circulating MicroRNAs (Ci-MiRNAs) in Tumor Reprogramming

Interactions between cancer cells and the TME usually occur via cytokines, hormones, growth factors and secreted microRNAs. Pathway analysis of the genes targeted by miRNAs in the greater part of tumors positively correlated with miRNA-mediated gene regulation among stromal cells, mainly the myofibroblast phenotype and vascular smooth muscle cells inhabiting the tumor space [16]. Sequentially, cancer-associated fibroblasts (CAFs) also regulate the TME and tumor cells by secreting microRNAs encapsulated as endosomal vesicles, which strongly promote the development of an aggressive breast cancer cell phenotype [87]. A differential expression profile identified miRs-21, -378e and -143 to be augmented in exosomes from CAFs in comparison to normal fibroblasts and this induced stemness and an EMT phenotype, promoting the development of an aggressive breast cancer cell phenotype [87]. The measures adopted by tumor cells to counter immune cell infiltration are via the secretion of tumoral exosomes that act as carriers of miRNAs, targeting mRNAs with a role in immune-suppressive pathways, the promotion of tumor cell communication, invasion, metastasis and induced drug resistance by targeting anti-apoptotic genes [88,89]. Tumor-cell-secreted microRNAs in the circulation may either be protected in micro-vesicles containing mature miRs or pre-miRs with RNA-induced silencing complexes (RISCs) or as exosome-free microRNA complexes with argonaute proteins or bound by high-density lipoprotein (HDL) [88]. Most often, the exosome acceptor/receiver cells would be the immediate stromal microenvironment, the immune and endothelial cells that adapts its responses to the needs of the transforming tumor clones. 

Metastatic human breast cancer cell lines secreted exosomes containing miR-200 that are absorbed by non-metastatic tumor cells and promote EMT and colonization at distant sites [90]. High plasma levels of miR-200c/141 are indicative of metastatic breast cancers rather than localized breast tumors, suggesting its potential role as a biomarker for detecting metastatic spread [88]. The influence of steroid hormones in modulating the tumor environment via microRNAs is evidenced wherein progesterone treatment and irradiation stimulated the expansion of radiation-resistant tumor-initiating CSC compartment followed by the downregulated expression of miR-22 and miR-29c both in (PR+) BC cells and in (PR-) normal cells [91]. MiR-9, miR-15b, miR-17, miR-19a and miR-30d are identified to be the most interconnected differentially expressed (DE) ci-miRs that are highly abundant in tumor interstitial fluids (TIFs) of the basal subtype than in patients with luminal and Her2-enriched cancer and are also suggestive of high-grade metastatic tumors with poor prognosis [88]. The TNBC subtype is known for a high immune score of tumor-infiltrating lymphocytes (TILs) and yet it is capable of modulating the scale of immunity vs. tolerance by reducing the expression of a panel of miRs, (miR-146a, miR-494, miR-206, miR-369 and miR-376a) that are linked to the immune system and are normally found to be co-abundant in the blood of BC patients [88]. Tumor-derived exosomes enable the differentiation of TME fibroblasts into cancer-associated fibroblasts (CAFs) that also share the wound healing properties of myofibroblasts in promoting tumor growth and pro-angiogenic, invasive and drug-resistant phenotypes [89]. MiR-21 is a highly expressed oncogenic microRNA in BC that promotes tumor cell survival and the formation of CAFs by regulating *TGFβ1* signaling [92]. MiR-155 and miR-210 are ci-oncomiRs highly expressed in BC and are pivotal in inducing metabolic changes in human adult fibroblasts, thereby promoting a pre-metastatic microenvironment [92,93]. Tumor-derived exosomes also polarize the macrophages in the TME to form tumor-associated macrophages (TAMs) that can bi-directionally modulate the TME into an anti-inflammatory, immunosuppressed state by releasing exosomes carrying miRNAs such as miR-21 and miR-223, thereby leading to enhanced drug resistance, suppression of cancer cell apoptosis and activation of the PI3K/AKT signaling pathway [93]. 

CSCs also impact multidrug resistance in BC through the overexpression of ATP-binding cassette (ABC) transporter proteins, ABCB1 (P-glycoprotein), ABCC1 and ABCG2 (BCRP1), which reduces the concentrations of chemotherapeutic drugs to suboptimal levels in the TME [93,94]. Doxorubicin-resistant cancer exosomes are observed to deliver six different miRNAs known to be associated with drug resistance (miR-204-5p, miR-139-5p, miR-29c-5p, miR-551b-3p, miR-29b-2-5p and miR-204-3p) [95]. In addition, miRNA-298 effectively targeted *ABCC1*, enhancing the sensitivity to DOX, whereas miRNA-328 and miRNA-487a amplified breast cancer cells’ sensitivity to mitoxantrone (MX) by directly targeting *ABCG2* [96]. Resistant tumor cells secrete exosomes incorporating ABC drug efflux pumps and P-glycoprotein in the cargo transport to neighboring sensitive cells, thereby magnifying the rate of malignant transformation in the TME [89]. Most often, exosomes carrying cargoes of oncomiRs are secreted from resistant tumor cells to sensitize cell fractions, influencing the cell transition to a more resistant version. Taking into account the multiple roles of microRNAs in inducing chemoresistance, it seems promising to utilize miRs in combination with anticancer drugs to reverse resistance and help to uncover actionable targets to improve the patient diagnosis/prognosis and disease monitoring. 

## 6. Conclusions

Heterogeneity between breast tumor subtypes, differential rates of pathological complete response (pCR) and distant relapse highlight the need to explore molecular determinants to accurately classify breast tumors and allow tracking of disease progression at different time points of therapy and subsequent identification of relapse [97]. Cataloguing mutations and copy number variations is an excellent method of identifying genomic differences between tumors, but may result in a loss of information regarding tumor lineage and disease progression. The different levels of survival mechanisms adopted by tumor cells commence at the molecular level, with small microRNAs acting as master regulators of tumor, stroma and immune cells in response to therapy [11,12,14,15,17]. The inherent stability in circulation and mobility to transform neighboring cells make microRNAs the best effectors of paracrine and autocrine signaling in promoting tumor progression [87]. MicroRNA profiling is apparently more robust than the current techniques such as IHC in discerning tumors that show clonal heterogeneity and for monitoring the emergence of recurrent disease of a different subtype than the primary tumor to justify a change in treatment option [11,12,13,14,15,16,17,18,19,20]. MicroRNAs are crucial in modulating the expression of multiple mRNAs, not through previously reported mechanisms of silencing and degradation, but by modes of co-activation and co-repression of both effector and target mRNAs [15]. Breast-cancer-associated miRNAs double up as markers of the residual disease post-surgical resection in the tissue and in the serum [3,17].

Through the consequential integration of data generated from the extensive screening of different molecular subtypes within a normal mammary tissue, and overlaying it with the data from mutations, proteomic analyses, phenogenomic cluster analysis and mathematical modeling of breast tumors, we have derived the conclusion that there are definitely ten breast epithelial subtypes with distinct molecular and phenotypic profiles with implications for diagnosis, prognosis and potential therapeutic strategy. This paper presents the molecular data befitting a translational model with microRNAs as key players that are present both in tissue and in circulation. There are several inconsistencies associated with (i) the selection of an ideal sample source: liquid biopsy (whole blood, plasma or serum) or fresh or paraffin-embedded solid tumor tissues; (ii) the preparatory methods for the quantitation of miRNAs and (iii) a universally accepted, appropriate reference miRNA as a normalizer to ensure the accurate comparability of results. The translational research would greatly benefit from the standardization and implementation of protocols to utilize these molecules routinely as prospective diagnostic or prognostic biomarkers in cancer patient care [43]. The intrinsic subtyping based on microRNAs from sample sources other than valuable tissues makes it even more lucrative as a prognostic molecular factor that could be tested using the basic quantitative polymerase chain reaction (qPCR) technique, processed within an average turn-around of less than 2 days, and is relatively cost-effective [98]. Elucidation of the microRNA-mediated molecular mechanisms that guide the prevalence of one tumor subtype over another will be the focus of our translational research and will allow personalized medicine to be developed in this fascinating arena. 

## Figures and Tables

**Table 1:** Differential expression of MicroRNAs in normal and cancer stem cells.

Tissue/Cell Type	List of MicroRNAs	Expression	MiRNA: Target Genes	Ref.
Normal mammary basal epithelial cell	miR-let7c, miR-125b, miR-126, miR-127-3p, miR-143, miR-145, miR-146-5p and miR-199a-3p	Upregulated	miR-145—*MUC1;* miR-125b—*AKT3*	[31,37]
Normal mammary luminal epithelial cell	miR-200c and miR-429	Upregulated	miR-200—*ZEB1, LGL2*	[31]
Normal mammary stem cells (MaSCs)	miRNA-200c-141, miR-200b-200a-429 and miR-183-96-182 cluster, miR-489	Downregulated	miR-200c/miR-200b/miR-429—*BMI1* miR-489—*HER2, DEK, SHP2*	[29,46]
Normal myoepithelial stem cells	miR-221	Upregulated	miR-221—*ATXN1, FNDC3A, ERBB3, PSD3*	[35]
MaSCs/BCSCs	miR-199a, miR-31, miR-196	Upregulated	miR-199a—*LCOR, Tox3, Rbm47;* miR-196—*HOXC8* miR-31—*Dkk1, Axin1, Gsk3β, Smad3, Smad4*	[34,41,45]
Luminal BCSC	miR-221	Upregulated	miR-221—*ATXN1*	[35]
Breast Cancer stem cells (BCSCs)	miRNA-200c-141, miR-200b-200a-429 and miR-183-96-182 cluster, miR-489	Downregulated	miR-200c/miR-200b/miR-429—*BMI1* miR-489—*HER2, DEK, SHP2*	[29,46]
Mature epithelial cells	miRNA-200c-141, miR-200b-200a-429 and miR-183-96-182 cluster	Upregulated	miR-200c/miR-200b/miR-429—*BMI1* miR-489—*HER2, DEK, SHP2*	[29]
Breast tumor cells	miR-10b, miR-125b, and miR-145	Downregulated	miR-10b—*FLT1, BDNF, SHC1* miR-125b—*YES, ETS1, TEL, AKT3, FGFR2, VTS58635,* miR-145—*MYCN, FOS, YES, FLI1, cyclin D2, CBFB*	[36]
Breast tumor cells	miR-17–5p, miR-29b–2, miR-181b–1, miR-146, miR-21 and miR-155	Upregulated	miR-17-5p—*E2F1;* miR-146—*ABL2, BCL11A* miR-21—*TGFB;* miR-155—*SOCS1, APC, WEE1, HIF1A*	[36,37]
Inflammatory breast cancer (IBC)	miR-221, miR-222, miR-18, miR-106B, miR-20	Upregulated	miR-221/222—*p27(Kip1)*	[40]
(IBC)/BCSCs	miR-141, miR-200a, miR-200b, miR-200c, miR-205, miR-335, Let-7 and miR-429	Downregulated	miR-200c—*ZEB1* miR-200b—*SIP1*	[39]
MaSCs	miR-93, miR-205 and miR-206	Upregulated	miR-205—*NOTCH2, NOTCH4, BMI1*, *MYC*, *NANOG*, *KLF4*, *OCT4*, *SOX2*, *SOX9* miR-206—*CORO1C, MXD4, TMSB4X, SFRP1, PTMA* miR-93—*JAK1, STAT3, AKT3, SOX4, EZH1, HMGA2*	[42,43,44]
DCIS (BCSCs)	miR-489, miR-140, miR-34	Upregulated Downregulated Downregulated	miR-489—*HER2*, *DEK, SHP2;* miR-140—*SOX9, ALDH1* miR-34—*NOTCH, p53, mTORC*	[46,47,48]

**Table 2:** An integrated phenotypic and transcriptomic characterization of breast tumor tissue.

No.	Cell Type	Receptor Phenotype (Hormone and Cytokeratin-Based)	Protein Phenotype	Tissue Subtype (Transcriptome-Based)	Integrated Cluster (CNA-Based)	Morphology
1	Tumor	HR+/CK7−	Phenotypes 31, 48, 53	Luminal A	3, 4+, 6, 7 and 8	Epithelial cells
2		HR low/CK low	Phenotype 31	Luminal A/B	6 and 8	Epithelial cells
3		HR+/Ki67+/CK7−/HER2+	Phenotype 33	Luminal B/HER2	9	Epithelial cells
4		HR+ (ER+)	Phenotype 31	Luminal B	6, 8 and 9	Epithelial cells
5		HR low/CK low	Phenotype 14	Luminal B		Epithelial cells
6		HR low/CK low	Phenotype 28	Luminal B	2 and 6	Epithelial cells
7		HR−/HER2+/CK7+/CK19+	Phenotype 46	Luminal B/HER2 enriched	3	Epithelial cells
8		HR−/Ki67+	Phenotype 57	Basal	4− and 10	Epithelial cells
9		HR−/basal CK+	Phenotype 51	Basal	4− and 10	Epithelial cells
10		HR−/CAIX+ (Hypoxia)	Phenotype 9	Basal	10	Epithelial cells
11		HR−/HER2+	Phenotype 16	HER2/Basal	4−, 5 and 10	Epithelial cells
12	Normal	HR+/CK7−	Phenotype 23 and 53	Normal mammary	3, 4− and 4+	Epithelial cells
13			Phenotype 23	Myoepithelial cell		Epithelial cells
14	Stromal cells	ER−/HER2−/SMA+	Phenotypes 11, 38, 34, 32, 12, 15, 55, 56, 2, 43, 44, 17 and 39	Myofibroblasts	10 and 4+	Fibroblast cells
15		ER+/Vim+/SMA−	Phenotypes 24, 37,30, 29, 21 and 3	Fibroblasts	9, 10 and 4+	Fibroblast cells
16		ER+	Phenotypes 8, 20, 27 and 52	Fibroblast-CD68+	8	Fibroblast cells
17		Desmin+/Vim+/SMA+	Phenotype 6	Vascular smooth muscle actin	3	Endothelial cell
18	Immune cells	Vim+Slug−	Phenotype 13	Non-lymphoid cells	10	Macrophage
19		ER+Vim+Slug+	Phenotype 19	Non-lymphoid cells	9, 10	Macrophage
20		Vim+CD45−/low	Phenotype 47	Non-lymphoid cells		Macrophage
21			Phenotypes 22, 41, 7 and 5	Lymphoid cells	9, 10	T cells
22		HER2+/ER−	Phenotype 36	Lymphoid cells	2 and 5	B cells

**Table 3:** Integrated molecular taxonomy of human mammary normal and tumor epithelial tissue. HR++ means < 50%; HR+ means < 20%; HR+++ means ≥ 50%.

Cellular Phenotype	Molecular Phenotype	HR0		HR1				HR2			HR3	Ref
		L1-2	L3	L4	L5	L6	L7	L8	L9	L10	L11	
Normal Luminal (HR/CK)	ER	-	-	+	-	-	-	+	+	-	+	[27]
	AR	-	-	-	+	-	-	+	-	+	+	
	VDR	-	-	-	-	+	+	-	+	+	+	
	CK5/14/17	-	+	-	-	-	+	-	-	-	-	
	CK7/8/18/Cld4	+	+	+	+	+	+	+	+	+	+	
	Ki67	+	-	-	-	-	-	-	-	-	-	
Normal Myoepithelial	My1/CD10+ SMA/p63	-	-	-	-	-	-	-	-	-	-	
	My2/CK5+	-	-	-	-	-	-	-	-	-	-	
Breast Tumors (HR)	ER	-	-	+	-	-	-	+	+	-	+	
	HER2	+	-	+	+	+	+	+	+	+	+	
	TNBC	+	+	-	+	+	+	-	-	+	-	
**Integrated Clusters**		**IC5**	**IC10**	**IC2**	**IC9**	**IC6**	**IC1**	**IC8**	**IC4**	**IC7**	**IC3**	[8]
Breast Tumor Epithelial Cells (Copy number variations)	Mutational Features	ERBB2 amplification	10p gain, 8q gain, 5q loss, 12p gain	11q13/14 amplification	8q gain, 20q amplification	8p12 amplification	17q23 amplification	1q gain, 16q loss	Devoid of CN alterations	16p gain, 16q loss, 8q amplification	Paucity of CN alterations	
	HR	HER2+++/ER++/PR+	ER+/PR+/HER2+	ER+++/PR+++/HER2+	ER+++/PR+++	ER+++/PR++	ER+/PR++/HER2+	ER+++/PR+++	ER+++/PR++	ER+++/PR+++	ER+++/PR+++	
	Clinical features	Younger age; LN+; High Grade	Younger age; High grade; Large tumors	No distinct features	Older age; Low grade	No distinct features	High grade	Older age; Low grade	Low grade	Older age; Low grade	Low grade, Low LN+	
	Prognosis	Poor	Poor	Poor	Intermediate	Intermediate	Intermediate	Good	Good	Good	Good	
**Proteomic Landscape**		**IC5**	**IC4-/IC10**	**IC2**	**IC9**	**IC6**	**IC1**	**IC8**	**IC4+**	**IC7**	**IC3**	[16]
Breast Tumor Epithelial Cells (Pheno- genomic study)	Receptor Phenotype	HR-/HER2+	HR-/Ki67+ HR-/HER2+/basal CK+; HR-/CAIX+ (Hypoxia)	HR low/CK low HR+/CK7−	HR+/CK7−/Ki67+ HR-/CK7− HR+/CK7−	HR low/CK low HR+/CK7−	-	HR+/CK7−	HR+/CK7−	HR+/CK7−	HR-/CK7+/HER2+ HR+/CK7− HR+/CK7−/Slug+	
	Phenotype (Hormone and cytokeratin)	Phenotype 16	Phenotypes 9, 16, 51, 57	Phenotypes 28, 31	Phenotypes 31, 33, 51	Phenotypes 28, 31	Tumor enriched in myofibroblast cells	Phenotypes 31, 48	Phenotypes 48, 53	Phenotypes 31, 48	Phenotypes 46, 48, 53, 54	
	%ER + %HER2+	53.0% 100%	0–20.0% 17.0–51.9%	94.7% 10.5%	87.1% 9.7%	100% 4.5%	96.0% 24.0%	100% 4.2%	100% 8.8%	97.1% 0%	96.4% 5.4%	
	Genomic Subtype (PAM50)	HER2+++/Basal	HER2+/Basal+++	LumA+++/LumB+	LumB	LumA+++/LumB+	LumA++/LumB++	LumA+++	LumA+++	LumA+++	LumA+++/LumB+	
	Integrated cluster (CNA)	5	4-, 10 and 5	2 and 6	9, 10	2 and 6	1	3, 4+, 6, 7 and 8	4+	3, 4+, 6, 7 and 8	3, 4+, 6, 7 and 8	
	Median Survival (% 5 year)	70.40%	73.20%	66.90%	60.60%	81.30%	78.30%	88.90%	98.00%	85.10%	92.30%	
	Stromal cells	Phenotype 36 B cells	Phenotype 23, 29, 30, 32, 5, 13 Myoepi, Myofb, Fb, Bcell, Macrophage (Vim+ Slug-)	Phenotype 36 B cells	Phenotype 5, 13, 19, 37 T-cell, Fb Macrophage (Vim+Slug-), Macrophage (Vim+Slug+)	-	Phenotype 42,55 Myofb	Phenotype 20 CD68+ Fb	Phenotype 55, 21 Myofb, Fb		Phenotype 38, 6, 21 SMA+/Myofb/Fb	
Topological Data Analysis	Re-classified PAM50 gene set	HER2/Basal	Basal/ Myoepithelial	Lum/HER2	LumB/Basal	LumA/Basal/ HER2	Myo/Lum/ HER2	Myo/LumA/ LumB	Luminal	Luminal	Myo/Lum/HER2	[5]
MicroRNA-based	Hormone receptor phenotype	Basal-HER2	Basal	HER2-LumB	Basal-LumB	Basal-LumA	HER2	LumB	LumA-LumB	LumA	HER2-LumA	[12]

## Abbreviations

miRs/MiRNAs—micro ribonucleic acids; BC—breast cancer; ROR—risk of relapse; ER—estrogen receptor; PR—progesterone receptor; ERBB2/HER2—human epidermal growth factor 2; TNBC—triple negative breast cancer; LA/LumA—Luminal A; LB/LumB—Luminal B; PAM50—Prediction Analysis of Microarray 50; TDA—Topological Data Analysis; Myo—Myoepithelial; IntClust/(ICs)—integrated clusters; CNAs—copy number alterations; HR+—hormone-receptor positive; HR-—hormone-receptor negative; DCIS—ductal carcinoma in situ; mRNA—messenger ribonucleic acid; ADH—atypical ductal hyperplasia; METABRIC—Molecular Taxonomy of Breast Cancer International Consortium; TCGA—The Cancer Genome Atlas; SCAN-B—The Sweden Cancerome Analysis Network-Breast initiatives; TDLUs—terminal duct lobular units; CSCs—cancer stem-like cells; VDR—vitamin D receptor; AR—androgen receptor; CK—cytokeratin; Cld—claudin; Ki-67 or MKI67—Marker of Proliferation Ki-67; MUC1—Mucin 1; MaSCs—mammary stem cells; CD—cluster of differentiation; BCSCs—breast cancer stem cells; EMT—epithelial–mesenchymal transition; MET—mesenchymal–epithelial transition; MRUs—mammary repopulating units; MaCFCs—mammary epithelial progenitor cells; IBC—inflammatory breast carcinoma; HBC—hereditary breast cancer; SBC—sporadic breast cancer; BRCAX—HBCs of unknown genetic origin; NBT—normal breast tissues; TME—tumor microenvironment; CAFs—cancer-associated fibroblasts; MAPKs—mitogen-activated protein kinases; MKPs—mitogen-activated protein kinase phosphatases; RFS—recurrence-free survival; DFS—disease-specific survival; CM—conditioned media; TZB—trastuzumab; RISCs—RNA-induced silencing complexes; HDL—high-density lipoprotein; DE—differentially expressed; TIF—tumor interstitial fluid; TIL—tumor-infiltrating lymphocyte; ci—circulatory; TAMs—tumor-associated macrophages; ABC—ATP-binding cassette transporter proteins; DOX—Doxorubicin; MX—mitoxantrone; pCR—pathological complete response.
